# Genetic Polymorphisms of UDP-Glucuronosyltransferases and Susceptibility to Antituberculosis Drug-Induced Liver Injury: A Systematic Review and Meta-Analysis

**DOI:** 10.1155/2023/5044451

**Published:** 2023-10-12

**Authors:** Xinyu Chen, Zhuolu Hao, Nannan Wang, Jia Zhu, Honggang Yi, Shaowen Tang

**Affiliations:** Department of Epidemiology and Biostatistics, School of Public Health, Nanjing Medical University, Nanjing, China

## Abstract

**Methods:**

The PRISMA statement was strictly followed, and the protocol was registered in PROSPERO (CRD42022339317). The PICOS framework was used: patients received antituberculosis treatment, UGTs polymorphisms (mutants), UGTs polymorphisms (wild), AT-DILI, and case-control studies. Eligible studies were searched through nine databases up to April 27, 2022. The study's qualities were assessed by the revised Little's recommendations. Meta-analysis was conducted with a random-effects model using odds ratios (ORs) with 95% confidence intervals (95% CIs) as the effect size.

**Results:**

Twelve case-control studies with 2128 cases and 4338 controls were included, and 32 single nucleotide polymorphisms (SNPs) in the seven UGT genes have been reported in Chinese and Korean. All studies were judged as high quality. The pooled results indicated that UGT1A1 rs3755319 (AC vs. AA, OR = 1.454, 95% CI: 1.100–1.921, *P* = 0.009), UGT2B7 rs7662029 (G vs. A, OR = 1.547, 95% CI: 1.249–1.917, *P* < 0.0001; GG + AG vs. AA, OR = 2.371, 95% CI: 1.779–3.160, *P* < 0.0001; AG vs. AA, OR = 2.686, 95% CI: 1.988–3.627, *P* < 0.0001), and UGT2B7 rs7439366 (C vs. T, OR = 0.585, 95% CI: 0.477–0.717, *P* < 0.0001; CC + TC vs. TT, OR = 0.347, 95% CI: 0.238–0.506, *P* < 0.0001; CC vs. TC + TT, OR = 0.675, 95% CI: 0.507–0.898, *P* = 0.007) might be associated with the risk of AT-DILI.

**Conclusions:**

The polymorphisms of UGT1A1 rs3755319, UGT2B7 rs7662029, and UGT2B7 rs7439366 were significantly associated with AT-DILI susceptibility. However, this conclusion should be interpreted with caution due to the low number of studies and the relatively small sample size.

## 1. Introduction

Tuberculosis (TB) is a chronic communicable disease caused by *Mycobacterium tuberculosis* that contributes to high morbidity and mortality worldwide. In 2021, an estimated 10.6 million people fell ill with TB worldwide [1]. TB is preventable and, in most cases, treatable. At least 85% of drug-susceptible TB patients are successfully treated [2]. However, anti-TB therapy is known to have a hepatotoxicity effect, and anti-TB drug-induced liver injury (DILI) (AT-DILI) has been a long-standing concern in the treatment of TB infection [3]. The reported incidence of AT-DILI varies widely from 2% to 28% in different countries, depending on the investigators' definition of DILI as well as the population being studied [4]. A significant upward trend in AT-DILI incidence was observed from 1999 to 2020 [5]. The clinical spectrum of AT-DILI includes asymptomatic elevation in liver tests to acute hepatitis and acute liver failure [6]. Prompt withdrawal of the anti-TB drugs is the most critical intervention in the management of AT-DILI [4], which could lead to treatment interruption and poor treatment outcomes [7]. Therefore, reducing the occurrence of AT-DILI is crucial for the control of TB.

Until now, the specific mechanisms associated with AT-DILI have been inadequately described. A number of hypotheses on the pathogenesis of AT-DILI have been proposed, such as drug metabolism and transport, immune response, oxidative stress, and mitochondrial dysfunction [8]. Many previous mechanistic studies have focused on the isoniazid (INH) metabolic pathway. However, rifampin (RIF) has been reported to cause hemolysis due to the production of drug-dependent antibodies [9]. It was believed that hemolysis can generate a larger amount of hemoglobin from destroyed erythrocytes in blood and result in increased levels of free heme [10]. In addition, cotreatment with RIF and INH also causes accumulation of the endogenous hepatotoxin protoporphyrin IX in the liver through the alteration of the heme biosynthesis pathway [11]. The principal product of heme catabolism, bilirubin, is eliminated by a conjugation reaction with glucuronic acid, and the glucuronidation reaction is mediated by uridine diphosphate (UDP)-glucuronosyltransferases (UGTs) [12]. The UGT family is a phase II enzyme group responsible for the glucuronidation of numerous endobiotics, xenobiotics, and drugs to facilitate their excretion from the body [13]. In humans, 19 functional UGT isoforms comprise two families (UGT1A and UGT2) based on genetic similarity [14]. For example, hydrophobic bilirubin is a toxic product of heme metabolism that can be transformed into hydrophilic bilirubin in the liver through conjugation with uridine diphosphate glucuronic acid under the action of UGT1A1 [15]. UGT1A1 dysfunction may lead to hepatic vulnerability induced by the accumulation of bilirubin in the liver [16]. Thus, the glucuronidation reaction accelerates the elimination of toxic compounds, which plays an important role in the development of AT-DILI.

Human UGT genes have a large number of genetic polymorphisms, which have been confirmed to modulate enzymatic activity or promoter activity [17], further affecting the individual genetic susceptibility to AT-DILI. Recently, a number of studies have investigated the association between single nucleotide polymorphisms (SNPs) in UGTs and the risk of AT-DILI. Among the UGTs, the UGT1A1 gene has been the most extensively studied, but with inconsistent results among different populations [18–21]. For example, the SNP rs4148323 AA genotype of UGT1A1 was found to significantly reduce the risk of AT-DILI in Chinese patients in one study [21], while it was not associated with AT-DILI in Chinese patients in another study [20]. In recent years, the relationships between SNPs in other UGT genes (UGT1A4 [22], UGT2B4 [3], and UGT2B7 [23]) and AT-DILI risk have also been reported. Therefore, it is necessary to conduct a comprehensive systematic review by retrieving all publications reporting the relationship between SNPs in UGT genes and the risk of AT-DILI and clarifying the pooled effects of polymorphisms for AT-DILI. In the present study, we summarize published data to evaluate the relationship between UGT polymorphisms and susceptibility to AT-DILI.

## 2. Materials and Methods

### 2.1. Search Strategy

This systematic review and meta-analysis was reported according to the PRISMA guidelines [24] and has been registered on PROSPERO with ID number CRD42022339317. A comprehensive literature search was performed in English databases (PubMed, Medline, Web of Science, Embase, and Cochrane Library) and Chinese databases (CNKI, WANFANG, VIP, and SinoMed) up to April 27, 2022. The Medical Subject Headings (MeSH) terms used in the search were “tuberculosis,” “antitubercular agents,” and “chemical and drug-induced liver injury.” Furthermore, the following MeSH synonyms, related terms, and free terms were also included: “antituberculosis,” “tuberculosis treatment,” “drug-induced liver injury,” “drug-induced hepatotoxicity,” “uridine diphosphate glucuronosyltransferase,” “UDP-glucuronosyltransferase,” “UGTs,” “uridine 5′-diphospho-glucuronosyltransferase,” “polymorphism,” and “variant.” The Boolean operators “and” and “or” were applied to combine these terms. At the same time, the reference lists of selected articles and relevant reviews were manually searched to gather other potentially eligible studies.

### 2.2. Eligibility Screening

Records identified through all searches were imported into the EndNote X9 software for screening studies, and duplicate records were removed. Two reviewers independently selected the relevant studies meeting the eligibility criteria by titles and abstracts. Full texts were referred to when the above information was inadequate or unspecific for the determination of eligibility. Any disagreement was discussed and resolved by consensus or by consulting a third reviewer.

The studies included in this research met the following eligibility criteria: (1) case-control studies designed to investigate the relationship between UGTs polymorphisms and AT-DILI; (2) all patients received anti-TB treatment, of which the case group had AT-DILI, while the control group did not have AT-DILI; (3) the genotype frequency data could be extracted and analyzed; and (4) the language was restricted to English or Chinese.

The exclusion criteria were the following: (1) conference abstracts, editorials, letters, case reports, reviews, and meta-analyses; (2) sample size for each group of less than 10; and (3) studies with repetitious data (the studies with the most recent or comprehensive data were selected).

### 2.3. Data Extraction

The following data were extracted from all included studies: (1) basic characteristics: the first author, publication year, and country of origin; (2) study characteristics: study design, sample size, diagnostic criteria of AT-DILI, method of causality assessment, treatment regimens, and genotyping method; (3) population characteristics: sex and mean age of total subjects; and (4) polymorphism results: genotype frequencies in AT-DILI cases and controls or adjusted odds ratios (ORs) with 95% confidence intervals (CIs) under different genetic models and covariates. The data extraction procedure was also performed independently by two reviewers. If there was any disagreement, it was resolved by a third reviewer. No authors were contacted for further information.

### 2.4. Quality Assessment

The study qualities were assessed by the revised Little's recommendations [25]. These criteria included seven items: (1) scientific design, (2) definite inclusion of study population, (3) explicit information on study population, (4) explicit diagnostic criteria on AT-DILI, (5) genetic detection method, (6) correct statistical analysis, and (7) logical discussion of study bias. Each item can be rated as “yes” (low risk of bias) or “no” (high risk of bias). One score was awarded if an item was judged as “yes.” Scores for all quality criteria were added together for an overall quality score, and a study score >4 was defined as high quality [26].

### 2.5. Statistical Analysis

The ORs and corresponding 95% CIs were calculated to identify the potential association between susceptibility to AT-DILI and UGTs polymorphisms. The statistical analysis strategies refer to previous literature [27]. Allele models (M vs. W) (W refers to a wild-type allele and M refers to a mutated allele), dominant models (MW + MM vs. WW), recessive models (MM vs. MW + WW), homozygote models (MM vs. WW), and heterozygote models (MW vs. WW) were employed to analyze their associations. The significance of the pooled effect size was determined by the *Z* test and Mantel–Haenszel random effects model, with *P* < 0.05 being considered statistically significant. The heterogeneity between studies was quantified by the Cochran *Q* test and the *I*^2^ statistic (*I*^2^ < 25%, low heterogeneity; *I*^2^ = 25–50%, moderate heterogeneity; and *I*^2^ > 50%, high heterogeneity) [28]. Subgroup analyses were performed by country of origin. Review Manager 5.4 software (Cochrane Collaboration, Nordic Cochrane Centre) was used for this meta-analysis.

## 3. Results

### 3.1. Study Identification and Characteristics

**Figure 1:**
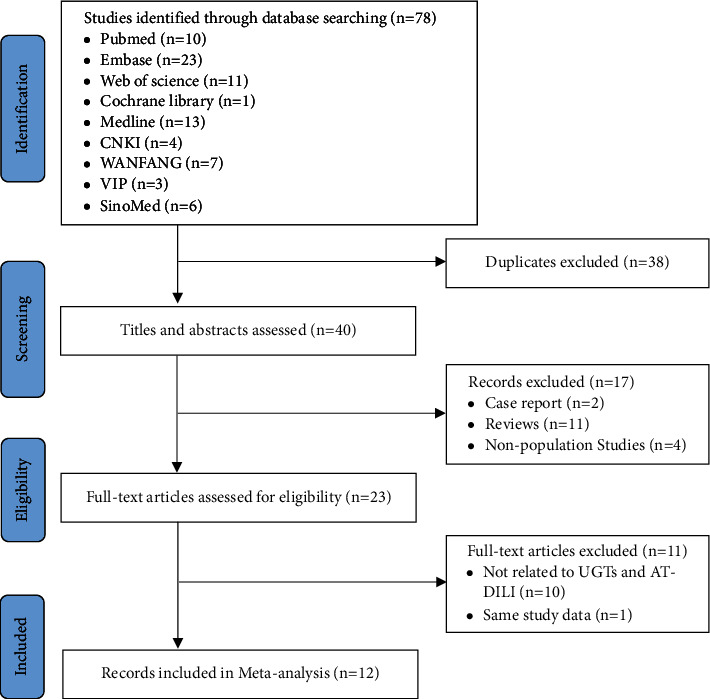
PRISMA flowchart for literature search. PRISMA, Preferred Reporting Items for Systematic Reviews and Meta-Analyses; UGTs, UDP-glucuronosyltransferases; DILI, drug-induced liver injury.

The flowchart for the selection of studies is presented in Figure 1. The initial search yielded 78 relevant records from the databases, and 40 records remained after disregarding duplicates. Then, 23 full texts were carefully assessed for eligibility after screening the titles and abstracts. Finally, 12 eligible studies describing the relationship between UGTs polymorphisms and susceptibility to AT-DILI were included in the present study [3, 15, 18–23, 29–32]. A total of 32 SNPs in the seven UGT genes (UGT1A1, UGT1A3, UGT1A4, UGT1A6, UGT1A7, UGT2B4, and UGT2B7) were reported in 12 studies (including 6466 patients (2128 AT-DILI cases and 4338 controls)). Eleven studies included participants of Chinese ethnicity [3, 15, 19–23, 29–32] and only one study included Korean participants [18]. The main anti-TB treatment regimen was a combination of first-line drugs (INH, RIF, pyrazinamide, and ethambutol with/without streptomycin). Only four studies performed causality assessment of AT-DILI [15, 20–22]. In terms of DILI criteria, 10 studies used alanine aminotransferase (ALT) > 2 upper limit of normal (ULN) [3, 15, 18–20, 22, 23, 29–31], one used ALT >3 ULN [32], and one used ALT >5 ULN [21]. The primary characteristics of the included studies are shown in Table 1. All studies were judged as high quality, and the average score was 6.4 (Supplementary Table 1).

### 3.2. Association of UGT1A1 Polymorphisms with AT-DILI

**Figure 2:**
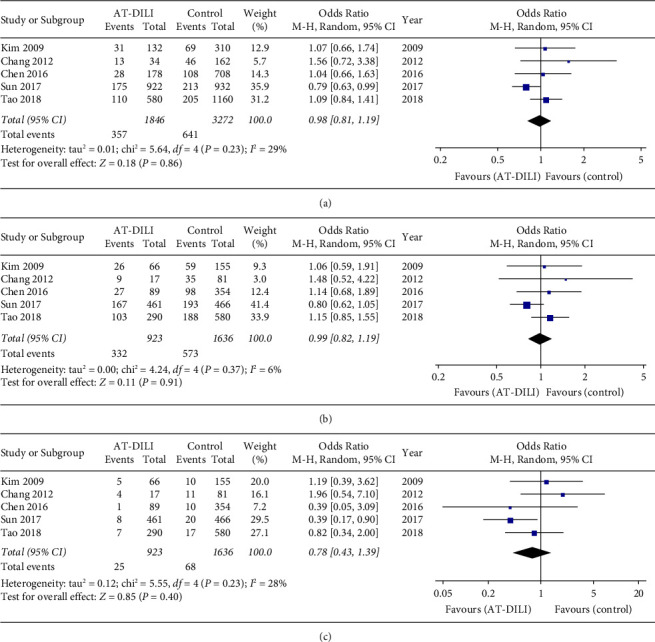
Forest plot of the relation between UGT1A1 SNP rs4148323 and AT-DILI risk with the random effects model. (a) Allele model. (b) Dominant model. (c) Recessive model. UGT1A1, UDP-glucuronosyltransferases 1A1; AT-DILI, antituberculosis drug-induced liver injury.

Among the seven UGT genes, the UGT1A1 gene was the most frequently reported; it was reported in 5 case-control studies [15, 18–21] including 924 cases and 1642 controls and 14 SNPs. All five studies analyzed SNP rs4148323, and the pooled result showed that it was not statistically associated with AT-DILI risk under any genetic model (allele model: A vs. G, OR = 0.983, 95% CI: 0.811–1.191, *P* = 0.857, Figure 2(a); dominant model: AG + AA vs. GG, OR = 0.989, 95% CI: 0.824–1.188, *P* = 0.909, Figure 2(b); and recessive model: AA vs. AG + GG, OR = 0.775, 95% CI: 0.432–1.391, *P* = 0.393, Figure 2(c), Table 2). A subgroup analysis of the Chinese population (four studies with 857 cases and 1483 controls) also did not find any association between SNP rs4148323 and AT-DILI risk (Tables 2 and 3, Supplementary Figure 1).

**Figure 3:**
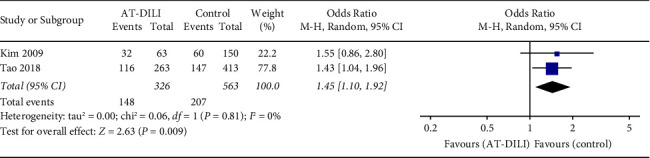
Forest plot of the relation between UGT1A1 SNP rs3755319 (heterozygote model) and AT-DILI risk with the random effects model. UGT1A1, UDP-glucuronosyltransferases 1A1; AT-DILI, antituberculosis drug-induced liver injury.

Four SNPs (rs2003569, rs8330, rs4148328, and rs3755319) were reported by two different studies [15, 18, 20, 21]. The pooled result showed that only one SNP (rs3755319) was associated with the risk of AT-DILI (heterozygote model: AC vs. AA, OR = 1.454, 95% CI: 1.100–1.921, *P* = 0.009, Figure 3 and Supplementary Figures 2, 3, 4, and 5). In addition, only a single Chinese study reported the relationship between nine SNPs (rs887829, rs35350960, rs8175347, rs34946978, rs4148326, rs12479045, rs11563250, rs6719561, and rs4148329) and AT-DILI risk [15, 19, 21] and only SNP rs6719561 was associated with a reduced risk of AT-DILI (heterozygote model: TC vs. TT, OR = 0.72, 95% CI: 0.53–0.99, *P* = 0.04) [15].

### 3.3. Association of UGT2B7 Polymorphisms with AT-DILI

**Figure 4:**
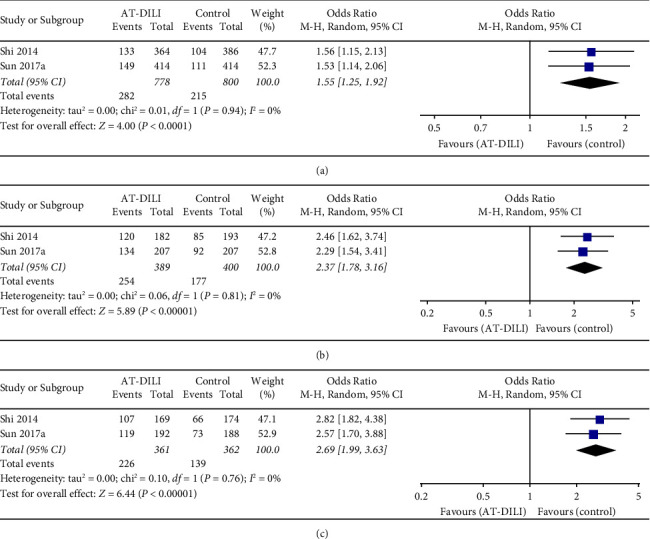
Forest plot of the relation between UGT2B7 SNP rs7662029 and AT-DILI risk with the random effects model. (a) Allele model. (b) Dominant model. (c) Heterozygote model. UGT2B7, UDP-glucuronosyltransferases 2B7; AT-DILI, antituberculosis drug-induced liver injury.

**Figure 5:**
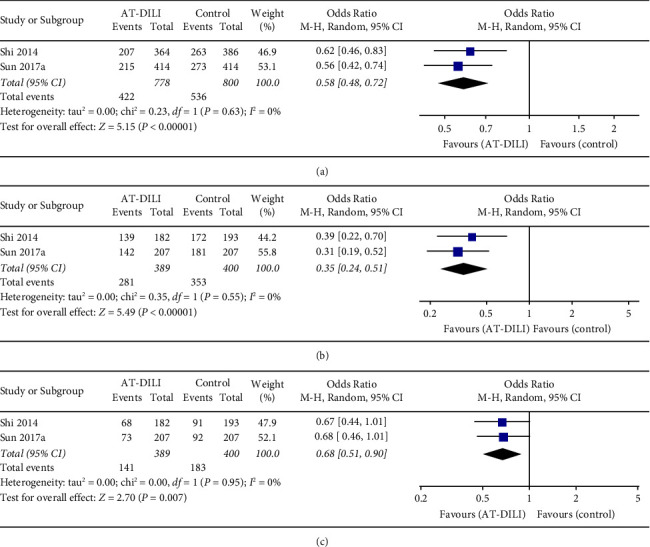
Forest plot of the relation between UGT2B7 SNP rs7439366 and AT-DILI risk with the random effects model. (a) Allele model. (b) Dominant model. (c) Recessive model. UGT2B7, UDP-glucuronosyltransferases 2B7; AT-DILI, antituberculosis drug-induced liver injury.

Three case-control studies with 413 cases and 623 controls focused on the relationships of UGT2B7 polymorphisms with AT-DILI in a Chinese population [23, 31, 32]. Four SNPs (rs7662029, rs7439366, rs10028494, and rs7668282) were reported, and the SNPs rs7662029 and rs7439366 were reported by two different studies [31, 32]. The pooled result showed that SNP rs7662029 was statistically associated with AT-DILI risk (allele model: G vs. A, OR = 1.547, 95% CI: 1.249–1.917, *P* < 0.0001, Figure 4(a); dominant model: GG + AG vs. AA, OR = 2.371, 95% CI: 1.779–3.160, *P* < 0.0001, Figure 4(b); and heterozygote model: AG vs. AA, OR = 2.686, 95% CI: 1.988–3.627, *P* < 0.0001, Figure 4(c), Tables 2 and 3). In addition, a significant association was also found between SNP rs7439366 and AT-DILI risk under all genetic models (allele model: C vs. T, OR = 0.585, 95% CI: 0.477–0.717, *P* < 0.0001, Figure 5(a); dominant model: CC + TC vs. TT, OR = 0.347, 95% CI: 0.238–0.506, *P* < 0.0001, Figure 5(b); and recessive model: CC vs. TC + TT, OR = 0.675, 95% CI: 0.507–0.898, *P* = 0.007, Figure 5(c), Tables 2 and 3). Sensitivity analysis confirmed these significant relationships after excluding the low-quality study. Another two SNPs (rs10028494 and rs7668282) reported in a single study were not significantly associated with AT-DILI risk [23].

### 3.4. Associations of Other Genes in the UGT Family with AT-DILI

Five other genes in the UGT family with 14 SNPs (UGT1A3: rs2008584 and rs6431625; UGT1A4: rs2011404; UGT1A6: rs6759892, 308C/A, and rs2070959; UGT1A7: rs17868323, rs17868324, and rs11692021; and UGT2B4: rs1131878, rs1966151, rs28361541, rs4557343, and rs79407331) were reported in five studies among Korean [18] or Chinese patients [3, 22, 29, 30]. Seven SNPs were associated with AT-DILI risk in Chinese anti-TB treatment patients. For example, patients carrying the CC genotype of rs2011404 in UGT1A4 were at a reduced risk of moderate or severe liver injury (OR = 0.293, 95% CI: 0.093–0.921, *P* = 0.036) [22]. Three SNPs in UGT1A6 were found to be associated with AT-DILI risk under the additive model (rs6759892: OR = 2.275, 95% CI: 1.492–3.470, *P* < 0.001; 308C/A: OR = 3.399, 95% CI: 2.185–5.287, *P* < 0.001; and rs2070959: OR = 2.342, 95% CI: 1.493–3.675, *P* < 0.001) [30]. Another three SNPs in the UGT1A7 gene were also significantly associated with AT-DILI risk under the additive model (rs17868323: OR = 1.747, 95% CI: 1.177–2.592, *P* = 0.006; rs17868324: OR = 2.391, 95% CI: 1.597–3.579, *P* < 0.001; and rs11692021: OR = 2.383, 95% CI: 1.523–3.729, *P* < 0.001) [29].

## 4. Discussion

The present study aimed to verify whether current evidence supports the relationship between UGTs polymorphisms and AT-DILI risk. Our meta-analysis included 12 case-control studies involving 32 SNPs in the seven UGT genes. Based on two original studies, the pooled results indicated that UGT1A1 rs3755319 (heterozygote model) might be associated with AT-DILI risk. In addition, UGT2B7 rs7662029 (allele model, dominant model, and heterozygote model) and rs7439366 (allele model, dominant model, and recessive model) were also statistically associated with AT-DILI risk. Therefore, genetic variants in UGT1A1 and UGT2B7 may have relationships with susceptibility to AT-DILI; thus, they have potential for use as biomarkers in the anti-TB treatment population.

However, the SNP rs4148323, which is the most studied SNP to date, was found to have no significant association with AT-DILI risk under any genetic model. Subgroup analysis also obtained similar negative results in Chinese patients (Figure 2, Tables 2 and 3). Further analysis found that only one original study indicated that patients with the A allele of rs4148323 in UGT1A1 had a lower risk of AT-DILI (A vs. G, OR = 0.371, 95% CI: 0.161–0.857, *P* = 0.020) [21]. Other original studies did not show any significant association between the SNP rs4148323 and AT-DILI risk [15, 18–20]. Although these studies were all designed as case-control studies, differences in sample size, diagnostic criteria, and adjusted covariates cannot be ignored. For example, one 1 : 1 matched case-control study was conducted in China with the largest sample size to date (461 cases and 466 controls) [21]. That study employed 5 ULN of ALT as a diagnostic criterion and did not adjust for covariates in the analysis, while others employed a 2 ULN criterion and adjusted for some covariates. Previous studies have found that the rs4148323 homozygous mutation and heterozygous mutation caused the enzymatic activity of UGT1A1 to decrease by 30–40% and 60–70%, respectively, and then significantly increased total bilirubin levels in vivo [33]. Among patients who received anti-hepatitis C virus drug treatment, the A allele of rs4148323 in UGT1A1 could be considered as a risk factor for drug-induced ALT elevation and liver injury [34]. Therefore, further studies are needed to confirm the association between SNP rs4148323 and AT-DILI risk, although our meta-analysis found no association based on the present studies.

For UGT1A1 rs3755319, in silico analysis indicated that the rs3755319 C allele might induce transcription binding changes and reduce UGT1A1 expression [35]. However, a significant association between SNP rs3755319 and AT-DILI risk was found under the heterozygote model. The haplotype TGG (rs3755319-rs2003569-rs4148323) in UGT1A1 was discovered to be associated with a marginally higher risk of ATLI (OR = 5.071, 95% CI: 1.007–25.531, *P* = 0.049) [15], and no association was observed between rs3755319 and RIF pharmacokinetics in South African patients with TB [36]. Therefore, SNP rs3755319 as a genetic risk marker was not robust enough according to our results, and more original studies are needed to confirm the above conclusion. For the other two statistically significant SNPs (UGT2B7 rs7662029 and rs7439366), the present meta-analysis under multiple genetic models and two original case-control studies observed that the AG genotype of rs7662029 and the TT genotype of rs7439366 in UGT2B7 increased the risk of AT-DILI [31, 32]. A previous study indicated that genetic polymorphisms in the coding and promoter regions of UGT2B7 had important clinical implications for pharmacology and toxicology and could induce AT-DILI through clinically significant changes in drug clearance [37]. This phenomenon has also been observed in other forms of DILI; for example, the T allele of rs7439366 in UGT2B7 was more common in diclofenac hepatotoxicity patients (OR = 8.5, 95% CI: 1.1–69.9, *P* = 0.026) [38]. Of course, determining the potential application value of rs7662029 and rs7439366 in UGT2B7 requires further research.

Identification of a genetic predisposition to AT-DILI is of paramount importance. These meta-analysis results indicated that UGT1A1 rs3755319, UGT2B7 rs7662029, and rs7439366 might be associated with the risk of AT-DILI, which would help to identify susceptible populations for liver injury in patients with anti-TB treatment. If used as a test prior to prescription, genotyping of these genes would prevent potential AT-DILI. However, although various genetic polymorphisms have been identified to be associated with DILI susceptibility, few prospective genetic screening tests have met the threshold for clinical application [39, 40]. The main reason is that the low incidence rate of DILI leads to a low positive predictive value for currently identified genetic variations, making them unsuitable for pre-prescription screening [41]. As described above, the reported incidence of AT-DILI is relatively low [4]. So, the low DILI incidence could not warrant the cost and effort associated with genetic testing [42]. Genetic polymorphisms of UGTs may be not useful in preemptive tests to reduce DILI incidence, but they can aid DILI diagnosis and clinical decision-making [40].

This study was the first to summarize all relevant studies investigating the relationships of UGTs polymorphisms with AT-DILI risk under different genetic models and to perform a meta-analysis of the data reported in those studies. The quality of the included studies was high. Nevertheless, the study had several limitations. First, the number of included studies was small, and the sample size was relatively small for determining genetic association, which made it difficult to draw a robust conclusion. Second, the study subjects were only Chinese and Koreans (limited to Asian countries), which minimized the possibility of discovering meaningful genetic associations. Because fewer than ten studies were included, a publication bias test was not performed. Finally, there existed a high heterogeneity for UGT1A1 rs4148328, and I-squares were larger than 60% under different genetic models. The diagnosis of AT-DILI, causality assessment, and adjustment for covariates were not uniform in those studies, which may be sources of potential heterogeneity.

## 5. Conclusion

The current meta-analysis indicated that UGT1A1 rs3755319, UGT2B7 rs7662029, and UGT2B7 rs7439366 were significantly associated with AT-DILI risk, and these three SNPs may be used as potential genetic risk markers in anti-TB treatment patients. However, this conclusion should be interpreted with caution due to the low number of studies and the relatively small sample size.

## Figures and Tables

**Table 1:** Characteristics of the included studies.

Genes	Study ID	Countries	Study design	Male (%)	Mean age (case/control)	Sample size (cases/controls)	Causality assessment	Diagnostic criteria	SNPs	Genotyping methods	Adjusted covariates	Treatment regimen used
UGT1A1	Kim 2009	Korea	Matched case-control study^a^	65.5	42.1/42.8	67/159	NR	>2 ULN	rs3755319, rs2003569, rs4148323	SNP-IT	Age, sex, baseline serum AST and ALT	HRZE
UGT1A1	Chang 2012	China, Taiwan	Case-control study	67.3	60.4/53.4	17/81	NR	>2 ULN	rs4148323, rs35350960, rs8175347, rs34946978	PCR-RFLP	NR	HRZE
UGT1A1	Chen 2016	China	1 : 4 matched case-control study^b^	73.0	43.7/43.6	89/356	WHO-UMC	>2 ULN	rs4148323, rs8330	TaqMan	Weight, hepatoprotectant use	HRZE(S)
UGT1A1	Sun 2017	China	1 : 1 matched case-control study^a^	61.5	38.2/38.9	461/466	RUCAM	>5 ULN	rs4148323, rs4148326, rs4148328, rs12479045	SNPscan	NR	HRZE
UGT1A1	Tao 2018	China	1 : 2 matched case-control study^b^	75.5	47.5/47.5	290/580	RUCAM	>2 ULN	rs2003569, rs887829, rs8330, rs3755319, rs4148328, rs11563250, rs4148323, rs6719561, rs4148329	Sequenom MassARRAY	Weight, hepatoprotectant use	HRZE(S)
UGT1A3	Kim 2009	Korea	Matched case-control study^a^	65.5	42.1/42.8	67/159	NR	>2 ULN	rs2008584, rs6431625	SNP-IT	Age, sex, baseline serum AST and ALT	HRZE
UGT1A4	Zhu 2021	China	1 : 4 matched case-control study^c^	72.3	49.4/50.3	202/808	RUCAM	>2 ULN	rs2011404	Sequenom Mass array	Smoking, drinking, hepatoprotectant use, liver diseases	HRZE(S)
UGT1A6	Hao 2011	China	Case-control study	80.0	47.1/45.0	202/239	NR	>2 ULN	rs6759892, 308C/A, rs2070959	PCR-RFLP	Smoking, drinking	2HRZS(E)/4HR
UGT1A7	Hao 2012	China	Case-control study	80.0	47.1/45.0	202/239	NR	>2 ULN	rs17868323, rs17868324, rs11692021	PCR-RFLP	Smoking, drinking	2HRZS(E)/4HR
UGT2B4	Chen 2021	China	Case-control study	59.6	49.4/50.3	118/628	NR	>2 ULN	rs1131878, rs1966151, rs28361541, rs4557343, rs79407331	iMLDR	NR	2HRZE/4HR
UGT2B7	Shi 2014	China	Case-control study	70.7	46.7/44.8	182/193	NR	>2 ULN	rs7662029, rs7439366	PCR-RFLP	Smoking, drinking	NR
UGT2B7	Sun 2017	China	1 : 1 matched case-control study^d^	NR	NR	207/207	NR	>3 ULN	rs7662029, rs7439366	PCR-RFLP	NR	2HRZS(E)/4HR
UGT2B7	Chen 2017	China	Case-control study	39.7	32.8/38.9	24/223	NR	>2 ULN	rs10028494, rs7668282	Sequenom Mass array	Age, sex, BMI, smoking	2HRZE/4HR

NR, not report; PCR-RFLP, polymerase chain reaction and restriction fragment length polymorphism; WHO-UMC, World Health Organization-Uppsala Monitoring Centre system; RUCAM, Roussel Uclaf causality assessment method; SNPs, single nucleotide polymorphisms; AST, aspartate aminotransferase; ALT, alanine aminotransferase; ULN, upper limit of normal; H, isoniazid; R, rifampicin; E, ethambutol; Z, pyrazinamide; S, streptomycin. ^a^Matched with age and sex. ^b^Matched with age (±5 years), sex, treatment history, disease severity, drug dosage, and place. ^c^Matched with age (±5 years), sex, and treatment history. ^d^Matched with sex and place.

**Table 2:** Meta-analysis results of the association between SNPs in UGT1A1/UGT2B7 and AT-DILI risk under the allele model.

Genes	Country	SNPs	Study numbers	Heterogeneity test			Overall effect	
				*χ* ^2^	*P*	*I* ^2^	OR (95% CI)	*P*
UGT1A1	All	rs4148323 (G> A)	5	5.64	0.228	29	0.983 (0.811–1.191)	0.857
	All	rs2003569 (G> A)	2	0.00	0.993	0	0.989 (0.784–1.247)	0.923
	All	rs8330 (C > G)	2	0.44	0.506	0	0.852 (0.681–1.065)	0.159
	All	rs4148328 (T > C)	2	5.66	0.017	82	0.988 (0.711–1.375)	0.945
	All	rs3755319 (A > C)	2	0.66	0.415	0	1.044 (0.852–1.280)	0.677
	China	rs4148323 (G> A)	4	5.40	0.145	44	0.982 (0.777–1.242)	0.881

UGT2B7	China	rs7662029 (A > G)	2	0.01	0.938	0	1.547 (1.249–1.917)	<0.001
	China	rs7439366 (T > C)	2	0.23	0.632	0	0.585 (0.477–0.717)	<0.001

UGT1A1, UDP-glucuronosyltransferase 1A1; UGT2B7, UDP-glucuronosyltransferase 2B7; AT-DILI, antituberculosis drug-induced liver injury; SNPs, single nucleotide polymorphisms; OR, odds ratio; 95% CI, 95% confidence interval.

**Table 3:** Meta-analysis results of the association between SNPs in UGT1A1/UGT2B7 and AT-DILI risk under different genetic models.

Genes	Country	SNPs	Study numbers	Dominant model					Recessive model					Homozygote model					Heterozygote model				
				Heterogeneity test			Overall effect		Heterogeneity test			Overall effect		Heterogeneity test			Overall effect		Heterogeneity test			Overall effect	
				*χ* ^2^	*P*	*I* ^2^	OR (95% CI)	*P*	*χ* ^2^	*P*	*I* ^2^	OR (95% CI)	*P*	*χ* ^2^	*P*	*I* ^2^	OR (95% CI)	*P*	*χ* ^2^	*P*	*I* ^2^	OR (95% CI)	*P*
UGT1A1	All	rs4148323 (G> A)	5	4.24	0.374	6	0.989 (0.824–1.188)	0.909	5.55	0.238	28	0.775 (0.432–1.391)	0.393	5.97	0.204	33	0.780 (0.419–1.452)	0.433	3.03	0.552	0	1.017 (0.851–1.216)	0.852
	All	rs2003569 (G> A)	2	0.00	1.000	0	0.970 (0.740–1.272)	0.824	0.01	0.929	0	1.090 (0.574–2.070)	0.793	0.01	0.927	0	1.075 (0.562–2.054)	0.828	0.00	0.994	0	0.956 (0.720–1.269)	0.756
	All	rs8330 (C > G)	2	0.71	0.399	0	0.827 (0.634–1.079)	0.161	0.06	0.813	0	0.908 (0.553–1.491)	0.730	0.00	0.956	0	0.824 (0.499–1.361)	0.450	0.90	0.342	0	0.825 (0.617–1.104)	0.196
	All	rs4148328 (T > C)	2	4.47	0.034	78	0.970 (0.638–1.476)	0.887	3.15	0.076	68	1.025 (0.634–1.658)	0.918	5.23	0.022	81	1.003 (0.509–1.973)	0.994	2.69	0.101	63	0.966 (0.685–1.361)	0.843
	All	rs3755319 (A > C)	2	0.36	0.551	0	1.258 (0.969–1.634)	0.084	0.01	0.932	0	0.637 (0.404–1.002)	0.051	0.02	0.882	0	0.737 (0.461–1.178)	0.203	0.06	0.813	0	1.454 (1.100–1.921)	0.009
	China	rs4148323 (G> A)	4	4.18	0.243	28	1.000 (0.791–1.265)	0.998	4.72	0.196	36	0.704 (0.347–1.426)	0.330	5.10	0.166	41	0.708 (0.333–1.505)	0.370	3.03	0.387	1	1.017 (0.842–1.227)	0.862

UGT2B7	China	rs7662029 (A > G)	2	0.06	0.813	0	2.371 (1.779–3.160)	<0.001	0.03	0.858	0	0.739 (0.444–1.231)	0.246	0.01	0.938	0	1.219 (0.715–2.077)	0.467	0.10	0.76	0	2.686 (1.988–3.627)	<0.001
	China	rs7439366 (T > C)	2	0.35	0.554	0	0.347 (0.238–0.506)	<0.001	0.00	0.950	0	0.675 (0.507–0.898)	0.007	0.11	0.739	0	0.338 (0.225–0.508)	<0.001	0.59	0.443	0	0.359 (0.238–0.540)	<0.001

UGT1A1, UDP-glucuronosyltransferase 1A1; UGT2B7, UDP-glucuronosyltransferase 2B7; AT-DILI, antituberculosis drug-induced liver injury; SNPs, single nucleotide polymorphisms; OR, odds ratio; 95% CI, 95% confidence interval.

## Data Availability

The data supporting the findings of this study are available within the main manuscript and the supplemental files.

## Abbreviations

ALT: Alanine aminotransferase
AT-DILI: Antituberculosis drug-induced liver injury
DILI: Drug-induced liver injury
MeSH: Medical Subject Headings
INH: Isoniazid
RIF: Rifampin
SNPs: Single nucleotide polymorphisms
TB: Tuberculosis
UDP: Uridine diphosphate
ULN: Upper limit of normal
UGTs: UDP-glucuronosyltransferases.

## Supplementary Materials

Supplementary Materials Supplementary materials are given in the .docx file format. Supplemental Table 1: quality score of the studies included in the meta-analysis. Supplementary Figure 1: forest plot of the relation between SNP rs4148323 and the risk of AT-DILI in Chinese population. Supplementary Figures 2–5: forest plot of the relation between SNP rs3755319, SNP rs8330, SNP rs2003569, and SNP rs4148328 and the risk of AT-DILI with the random effects model.
