# Supplementary material for: Genetic Polymorphisms of UDP-Glucuronosyltransferases and Susceptibility to Antituberculosis Drug-Induced Liver Injury: A Systematic Review and Meta-Analysis
Source: J Trop Med. 2023 Oct 12;2023:5044451. doi: 10.1155/2023/5044451 (PMC10586897; doi:10.1155/2023/5044451)
Supplement: Supplementary Materials — are given in the .docx file format. Supplemental Table 1: quality score of the studies included in the meta-analysis. Supplementary Figure 1: forest plot of the relation between SNP rs4148323 and the risk of AT-DILI in Chinese population. Supplementary Figures 2–5: forest plot of the relation between SNP rs3755319, SNP rs8330, SNP rs2003569, and SNP rs4148328 and the risk of AT-DILI with the random effects model. [file 5044451.f1.docx]

Supplementary Table 1: Quality score of the studies included in the meta-analysis

|  | Scientific design | definite inclusion of study population | explicit information on study population | explicit diagnostic criteria on AT-DILI | genetic detection method | correct statistical analysis | logical discussion of study bias | score |
| --- | --- | --- | --- | --- | --- | --- | --- | --- |
| Kim 2009 | 1 | 1 | 1 | 1 | 1 | 1 | 0 | 6 |
| Hao 2011 | 1 | 1 | 1 | 1 | 1 | 1 | 0 | 6 |
| Hao 2012 | 1 | 1 | 1 | 1 | 1 | 1 | 0 | 6 |
| Chang 2012 | 1 | 1 | 1 | 1 | 1 | 1 | 1 | 7 |
| Shi 2014 | 1 | 1 | 1 | 1 | 1 | 1 | 0 | 6 |
| Chen 2016 | 1 | 1 | 1 | 1 | 1 | 1 | 1 | 7 |
| Sun 2017 | 1 | 1 | 1 | 1 | 1 | 1 | 0 | 6 |
| Sun 2017 | 1 | 1 | 0 | 1 | 1 | 1 | 0 | 5 |
| Chen 2017 | 1 | 1 | 1 | 1 | 1 | 1 | 1 | 7 |
| Tao 2018 | 1 | 1 | 1 | 1 | 1 | 1 | 1 | 7 |
| Chen 2021 | 1 | 1 | 1 | 1 | 1 | 1 | 1 | 7 |
| Zhu 2021 | 1 | 1 | 1 | 1 | 1 | 1 | 1 | 7 |

AT-DILI, anti-tuberculosis drug-induced liver injury.

a


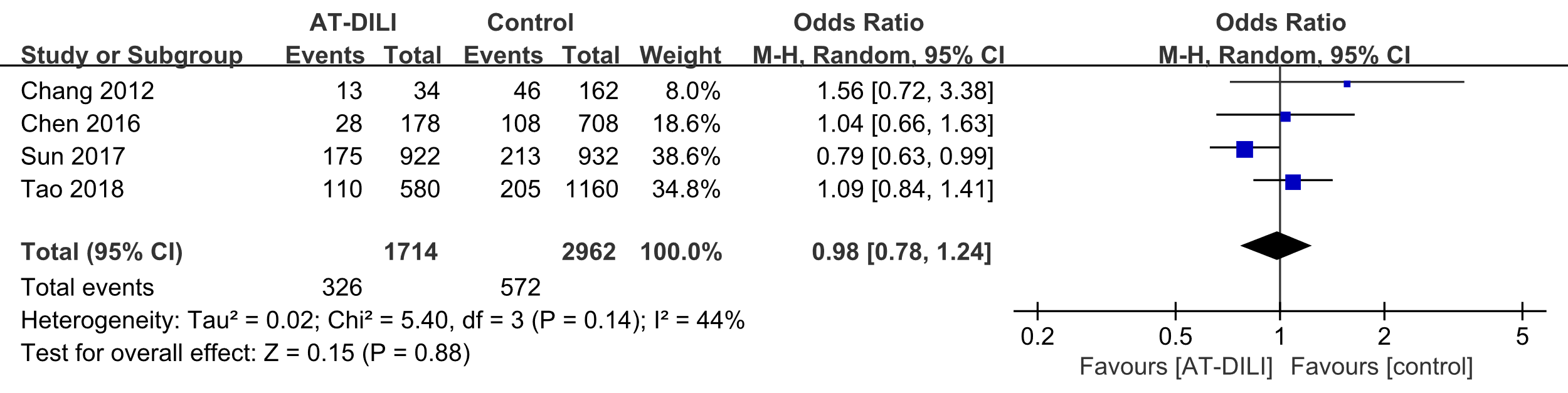


b
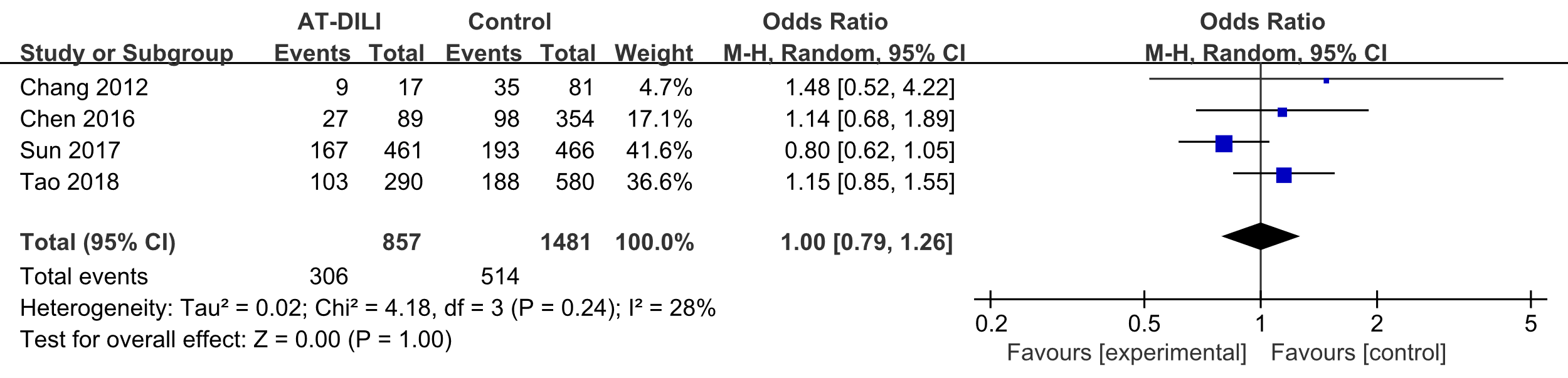


c


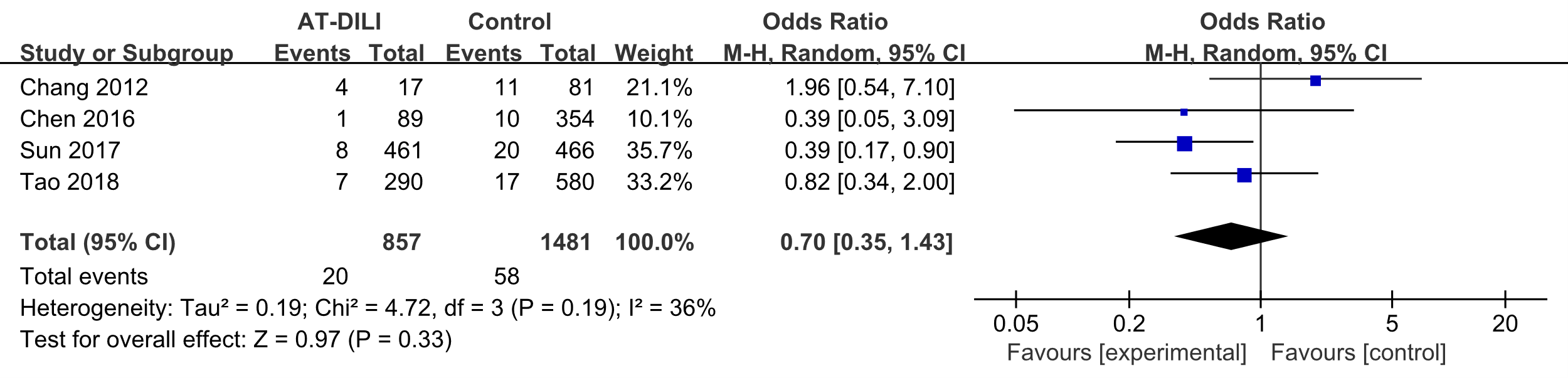


Supplementary Figure 1: Forest plot of the relation between SNP rs4148323 and the risk of AT-DILI in Chinese population. (a) allele model. (b) dominant model. (c) recessive model.

a
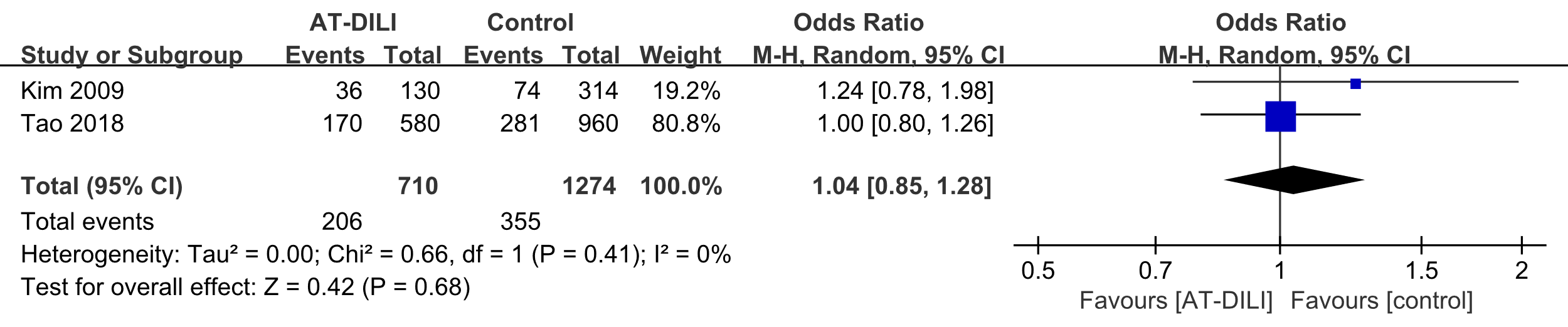


b
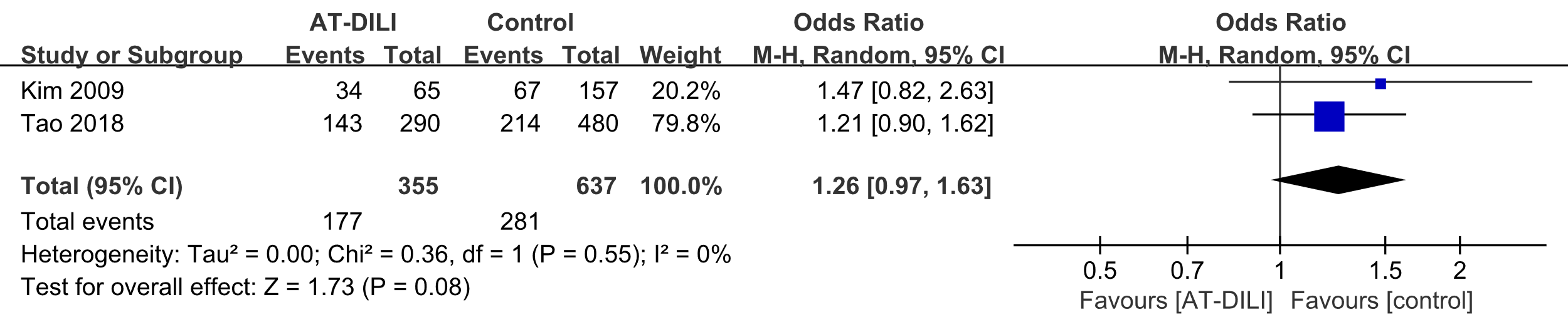


c
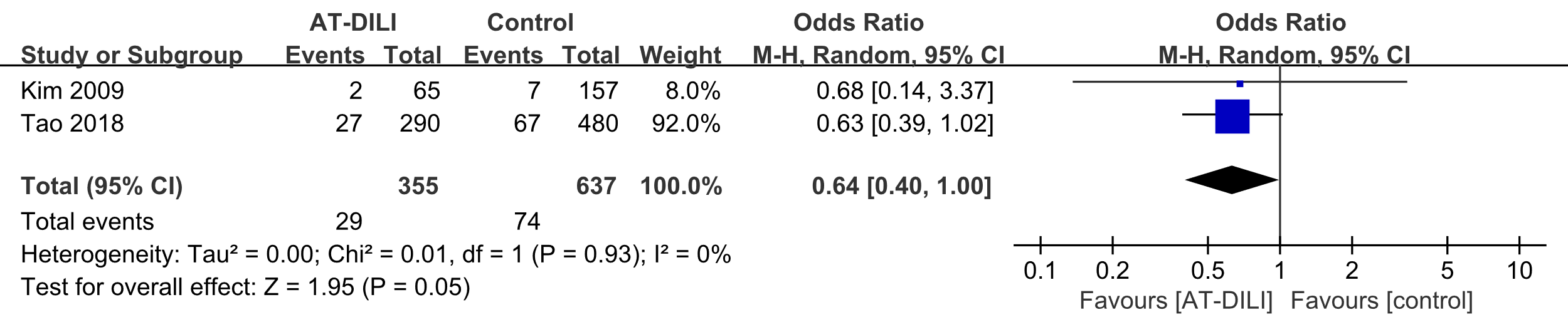


d
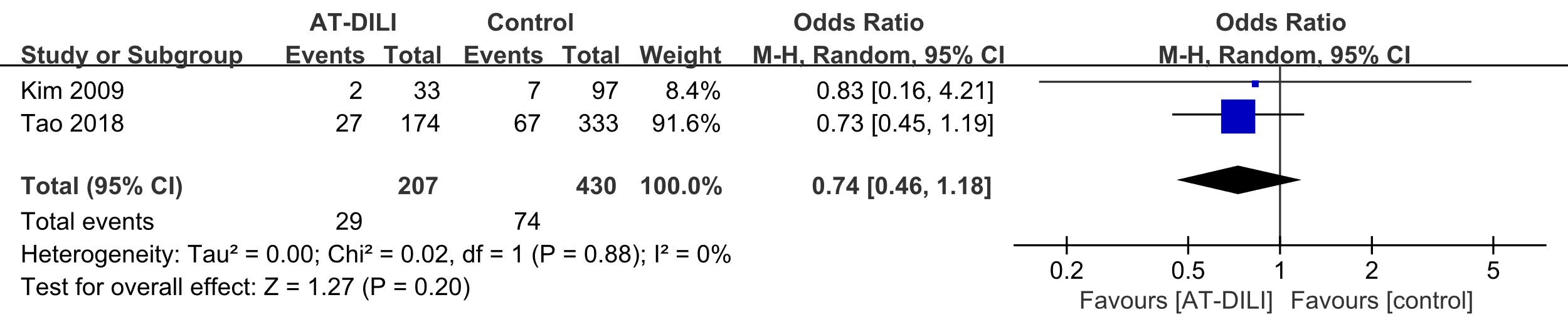


Supplementary Figure 2: Forest plot of the relation between SNP rs3755319 and the risk of AT-DILI with the random effects model. (a) allele model. (b) dominant model. (c) recessive model. (d) homozygote model.

a


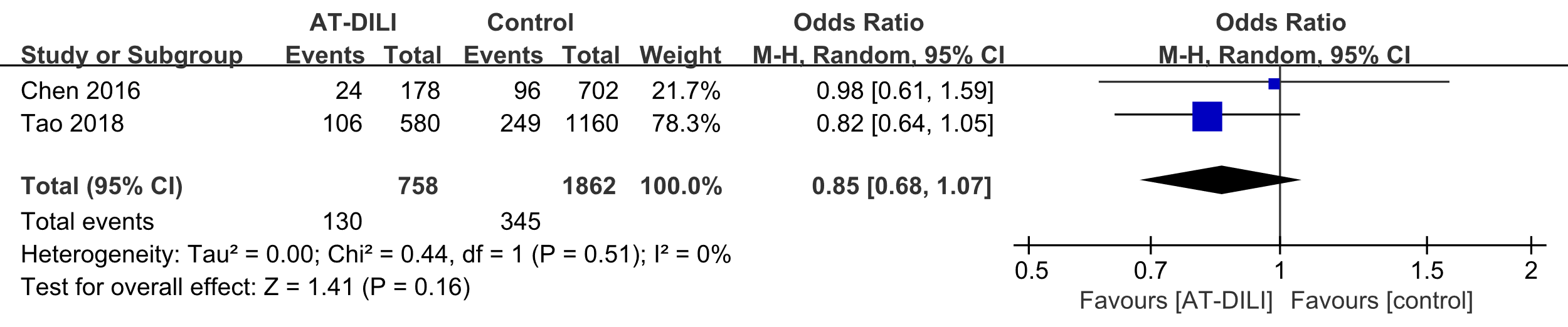


b


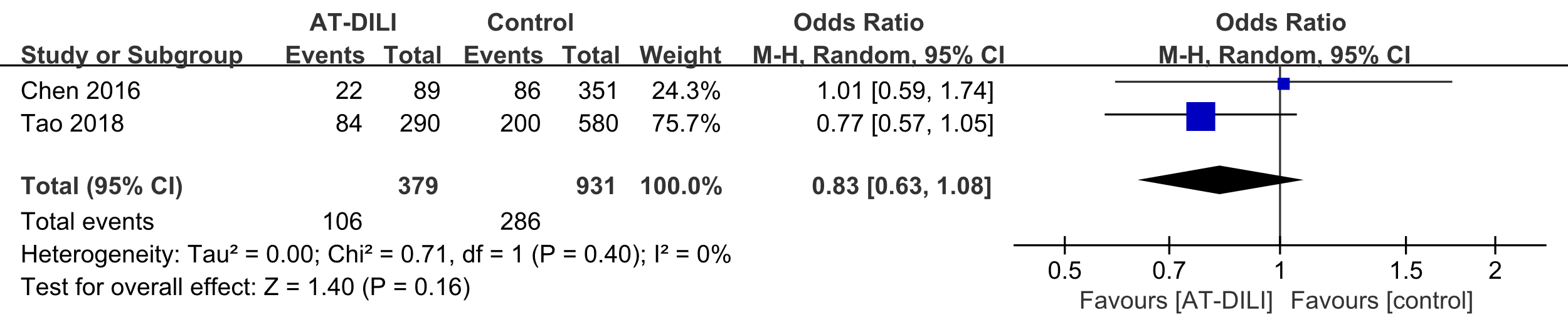
c


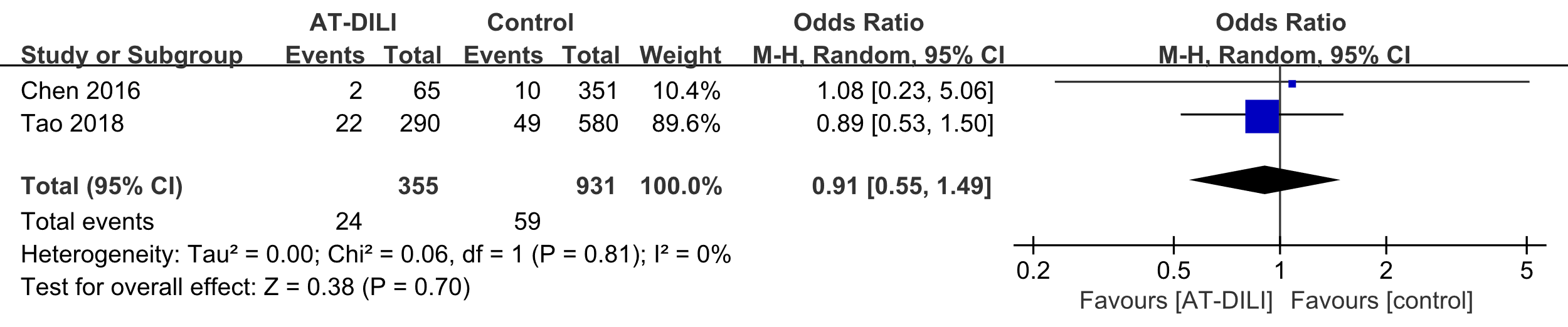


d


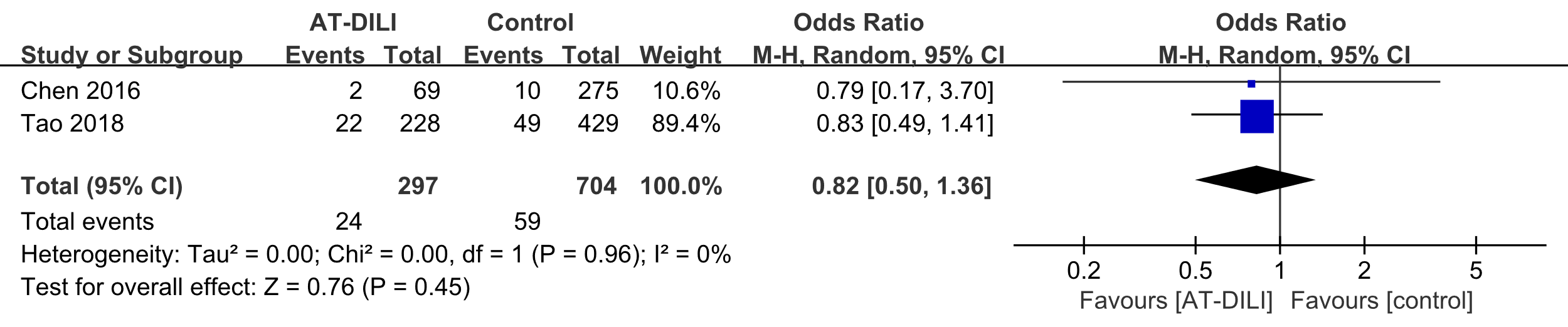


e


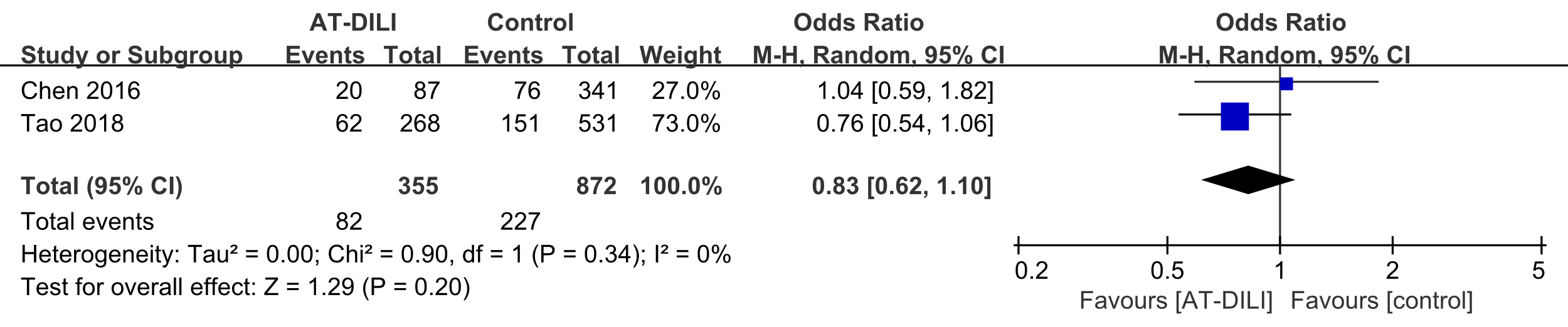


Supplementary Figure 3: Forest plot of the relation between SNP rs8330 and the risk of AT-DILI with the random effects model. (a) allele model. (b) dominant model. (c) recessive model. (d) homozygote model. (e) heterozygote model.

a


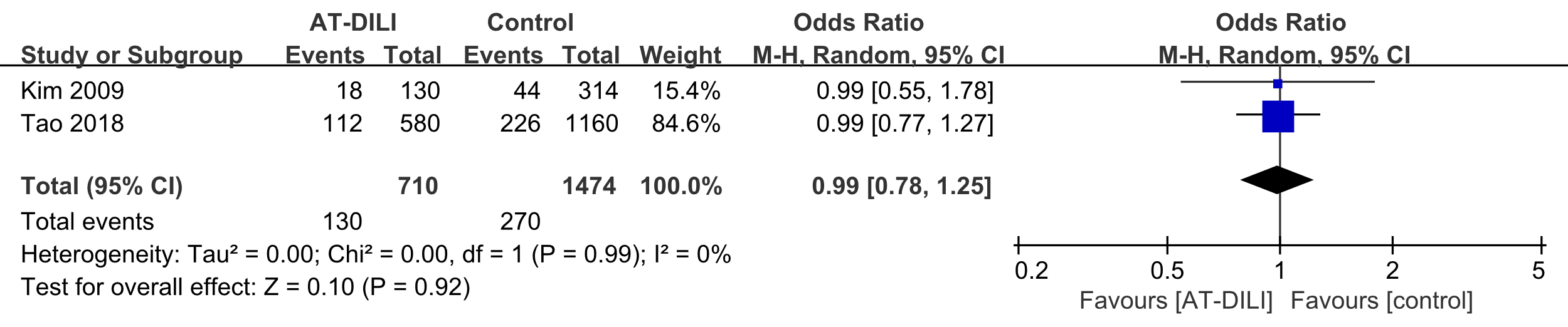


b


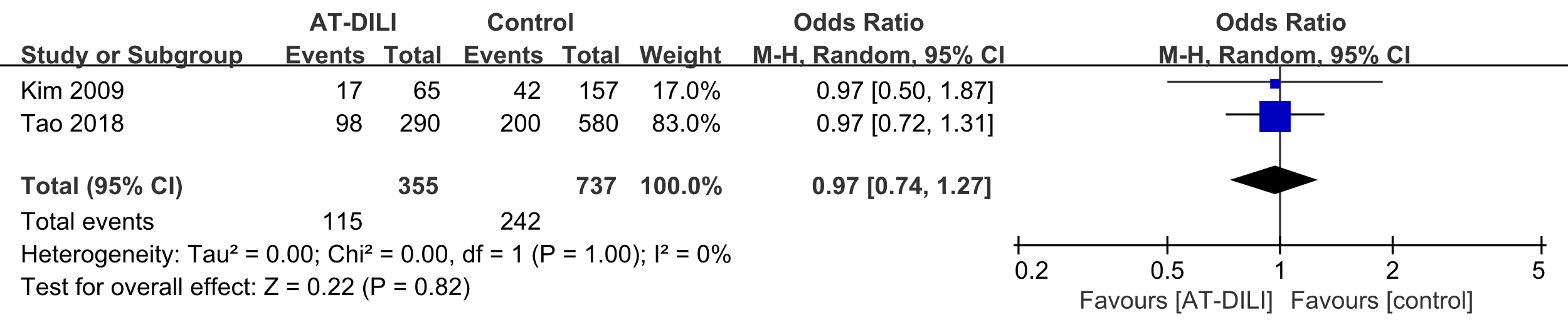


c


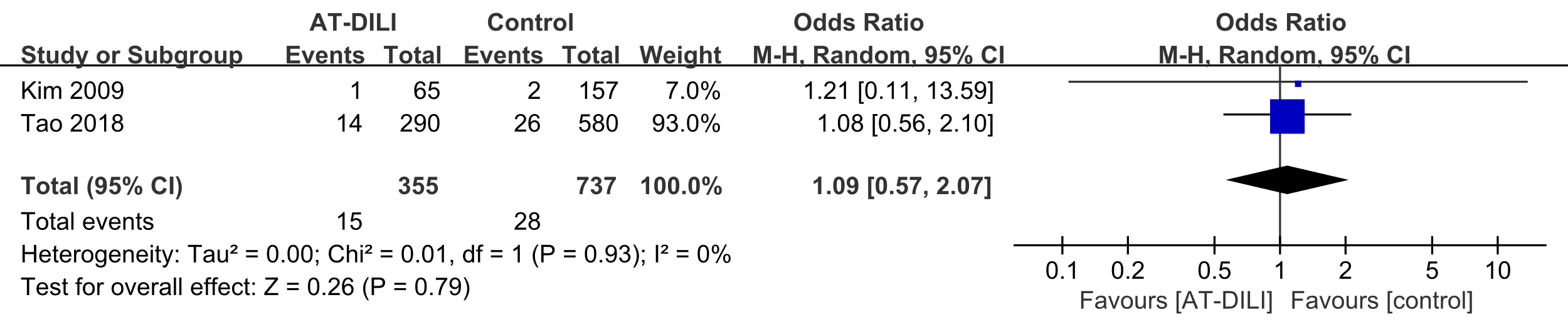


d


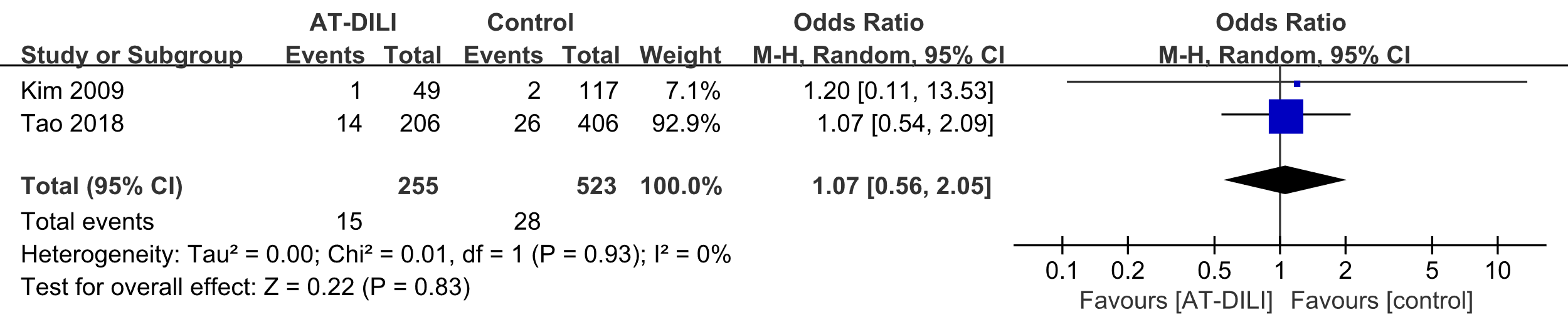


e


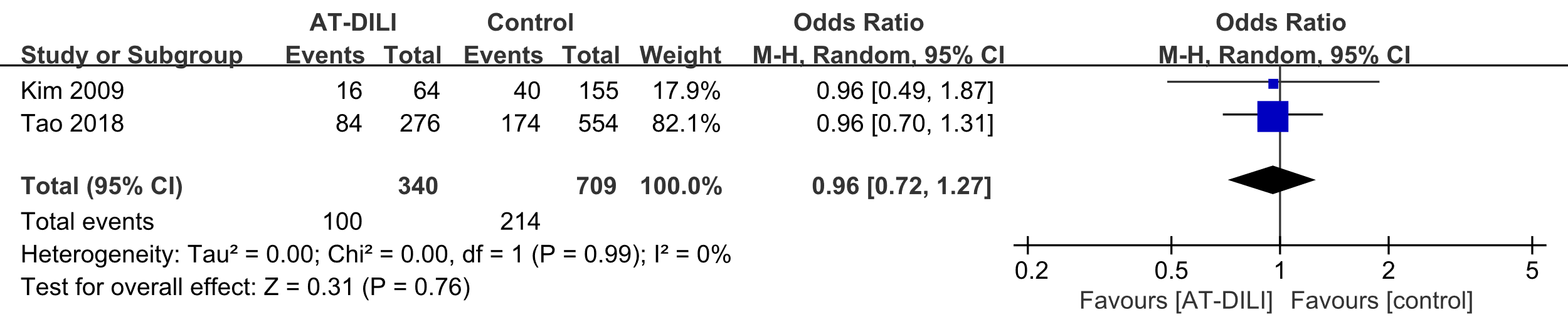


Supplementary Figure 4: Forest plot of the relation between SNP rs2003569 and the risk of AT-DILI with the random effects model. (a) allele model. (b) dominant model. (c) recessive model. (d) homozygote model. (e) heterozygote model.

a


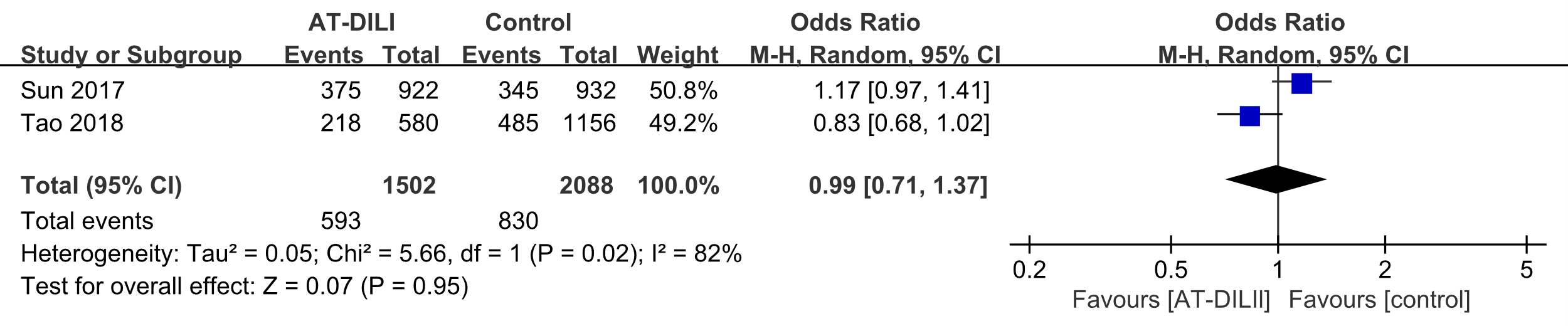


b


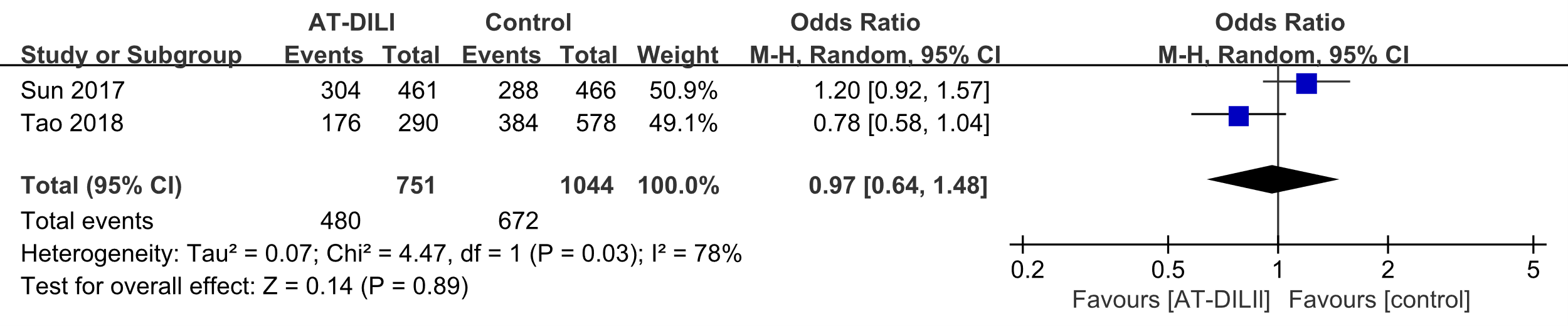


c


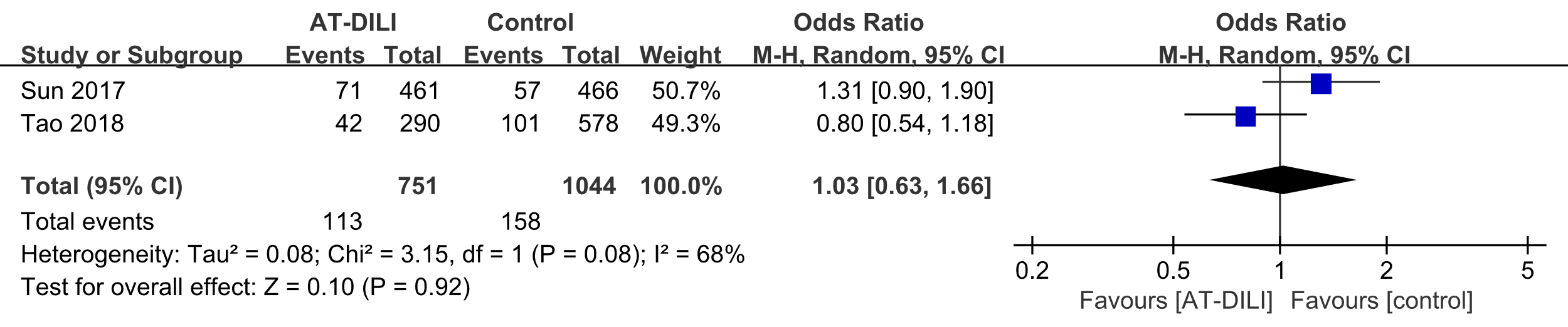


d


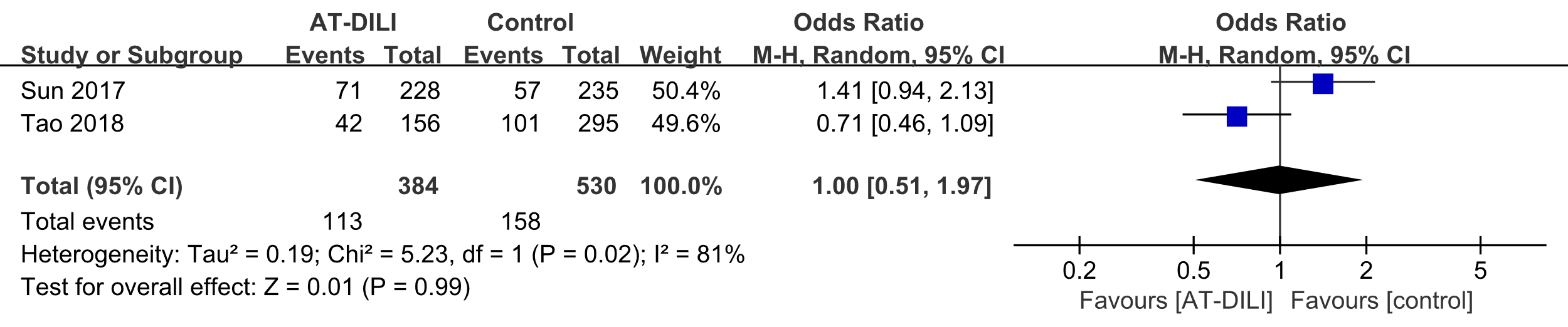


e


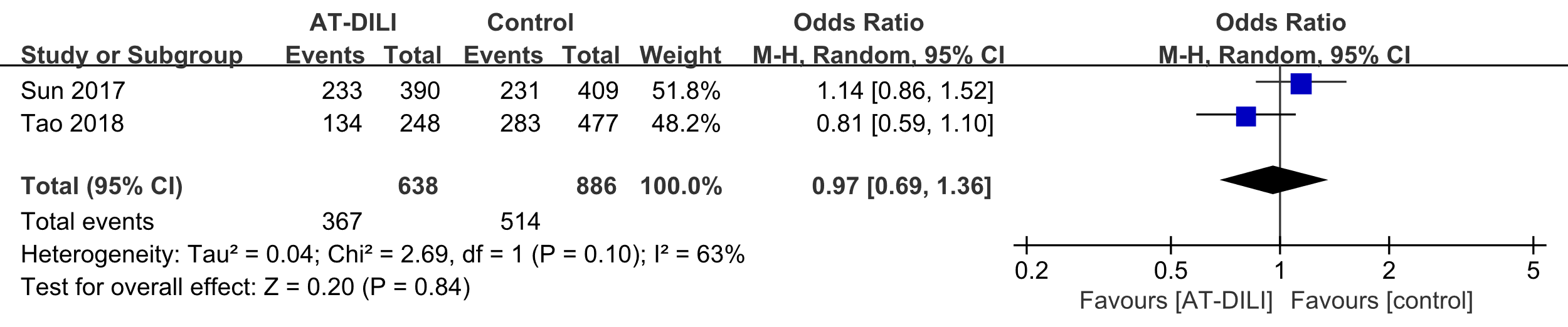


Supplementary Figure 5: Forest plot of the relation between SNP rs4148328 and the risk of AT-DILI with the random effects model. (a) allele model. (b) dominant model. (c) recessive model. (d) homozygote model. (e) heterozygote model.
